# Development of a Glycerol-Inducible Expression System for High-Yield Heterologous Protein Production in Bacillus subtilis

**DOI:** 10.1128/spectrum.01322-22

**Published:** 2022-08-29

**Authors:** Laichuang Han, Qiaoqing Chen, Jie Luo, Wenjing Cui, Zhemin Zhou

**Affiliations:** a School of Biotechnology, Jiangnan University, Wuxi, Jiangsu, China; University Roma Tre

**Keywords:** glycerol-inducible expression system, *Bacillus subtilis*, antiterminator protein, nattokinase, hybrid promoter

## Abstract

The development of efficient, low-cost, and robust expression systems is important for the mass production of proteins and natural products in large amounts using cell factories. Glycerol is an ideal carbon source for large-scale fermentation due to its low cost and favorable maintenance of the fermentation process. Here, we used the antiterminator protein GlpP and its target promoter P_glpD_ to construct a highly efficient glycerol-inducible expression system (GIES) in Bacillus subtilis. This system was able to express heterologous genes in an autoinducible manner based on the sequential utilization of glucose and glycerol under the regulation of carbon catabolite repression. In such a system, the concentration of glycerol regulated the strength of gene expression, and the concentration of glucose affected both the timing of induction and the strength of gene expression. By enhancing GlpP, the GIES was further strengthened for high-level intracellular expression of aspartase and secretory expression of nattokinase. High yields of nattokinase in a 5-L fermenter through batch and fed-batch fermentation demonstrated the potential to apply the GIES for large-scale enzyme production. Through the evolution of the −10 box of P_glpD_, mutants with gradient activities were obtained. In addition, hybrid glycerol-inducible promoters were successfully constructed by combining the constitutive promoters and the 5′ untranslated region of P_glpD_. Collectively, this study developed a GIES to obtain high-value products from inexpensive glycerol. More importantly, the great potential of the pair of inherent terminator and antiterminator protein as a portable biological tool for various purposes in synthetic biology is proposed.

**IMPORTANCE** In this study, a GIES was constructed in B. subtilis by employing the antiterminator protein GlpP and the GlpP-regulated promoter P_glpD_. Based on the sequential utilization of glucose and glycerol by B. subtilis, the GIES was able to express genes in an autoinducible manner. The amounts and ratio of glucose and glycerol can regulate the gene induction timing and expression strength. The GIES was further applied for high yields of nattokinase, and its robustness in production scale-up was confirmed in a 5-L fermenter. The high-level expression of heterologous proteins demonstrated the huge application potential of the GIES. Furthermore, mutants of P_glpD_ with gradient activities and hybrid glycerol-inducible promoters were obtained through the evolution of the −10 box of P_glpD_ and the combination of the constitutive promoters and the 5′ untranslated region of P_glpD_, respectively. These results demonstrated the use of the antiterminator protein as a regulator for various purposes in synthetic biology.

## INTRODUCTION

Efficient and precise expression of a gene of interest (GOI) has been a longstanding and essential research area for the construction of biological systems required for enzyme production, natural product synthesis, and synthetic biology (1–3). The GOI is expressed in either constitutive or inducible systems (4–6). Constitutive systems are unable to modulate the strength and timing of the GOI expression, making it difficult to rationally allocate limited resources between cell growth and product synthesis. On the other hand, inducible systems are able to decouple cell growth from GOI expression (7, 8), with autoinducible systems allowing cells to switch automatically from growth mode to production mode (9). The autoinducible expression of a GOI is often achieved in two ways. The first is a quorum-sensing system in which signaling molecules are synthesized during cell growth, and the GOI expression is activated or repressed when their concentration reaches a threshold (10, 11). The second is based on an inducible expression system whereby the inducer automatically functions during cell growth to regulate GOI expression. For example, Escherichia coli preferentially utilizes glucose; therefore, by adding both glucose and lactose to the medium, the widely used pET series vectors can express GOIs in an autoinducible manner (12).

Promoters that are inducible by carbon metabolites to initiate gene expression with high activity and specificity are widely used to construct inducible expression systems. These promoters include the xylose-inducible P_xylA_, maltose-inducible P_glv_, and methanol-inducible P_AOX1_ promoters (13–15). Moreover, such systems can easily be further developed into autoinducible expression systems simply by adjusting the medium components, which are tightly regulated by carbon catabolite repression (CCR). Among the various carbon sources, glycerol is ideal in the late stage of fermentation. Using glycerol instead of glucose greatly reduces the production of acetic acid during the fermentation process, which in turn reduces host stress during the late stage of fermentation while improving the utilization of carbon sources (16, 17). More importantly, glycerol is a by-product produced by biodiesel, fatty acid, detergent, and bioethanol industries. Hence, the abundance of low-value glycerol urgently needs to be transformed into high-value-added products (18, 19). In addition, the low price of glycerol reduces the cost of fermentation while avoiding potential food security problems with the use of carbon sources such as glucose and starch (20).

Bacillus subtilis is a generally recognized as safe (GRAS) microorganism isolated from soil; due to its strong protein secretion capacity and well-established genetic manipulation system, it has been used as a powerful chassis cell to produce several kinds of heterologous proteins (21–23). In recent years, although a variety of expression systems have been developed in B. subtilis to facilitate high protein yields, some limitations of those systems include cost and potential toxicity issues (24–26). Hence, use of the B. subtilis expression system could be beneficial for large-scale expression of molecules of interest if the cost and/or toxicity of the inducers to the hosts could be further reduced. B. subtilis is able to effectively utilize glycerol as a carbon source, benefiting from the presence of the *glp* operon gene products responsible for efficient glycerol metabolism. GlpP, the glycerol uptake operon antiterminator regulatory protein, is the vital regulator in the glycerol-inducible *glp* operon. After binding the ligand glycerol-3-phosphate (G3P), GlpP activates the expression of five genes (*glpD*, *glpF*, *glpK*, *glpQ*, and *glpT*) by disrupting the terminator located immediately downstream of the promoter (27–29). Recently, molecular docking and molecular dynamic simulations were used to resolve the structure-based mechanism and allostery of GlpP binding to G3P. (30).

Here, we developed a glycerol-inducible expression system (GIES) using the *glpD* promoter (P_glpD_), with higher activity than the strong constitutive promoter P43 (31). The GIES was highly robust in different genetic environments. Taking into account the regulation mediated by CCR, autoinduction of heterologous protein expression by the GIES was achieved through complexing of glucose and glycerol. By overexpression of *glpP*, the GIES was further enhanced for high-level production of aspartase (AspA) and nattokinase (NK). The potential for the GIES to be applied to large-scale enzyme production was demonstrated via the high yields of NK in a 5-L fermenter. Furthermore, mutants of P_glpD_ with gradient activity and hybrid glycerol-inducible promoters were obtained through the evolution of the −10 box of P_glpD_ and the combination of the constitutive promoters and the 5′ untranslated region (UTR) of P_glpD_, respectively. These results demonstrated the use of antiterminator protein as a regulator for various purposes in synthetic biology.

## RESULTS AND DISCUSSION

### Design, construction, and verification of the GIES.

**FIG 1:**
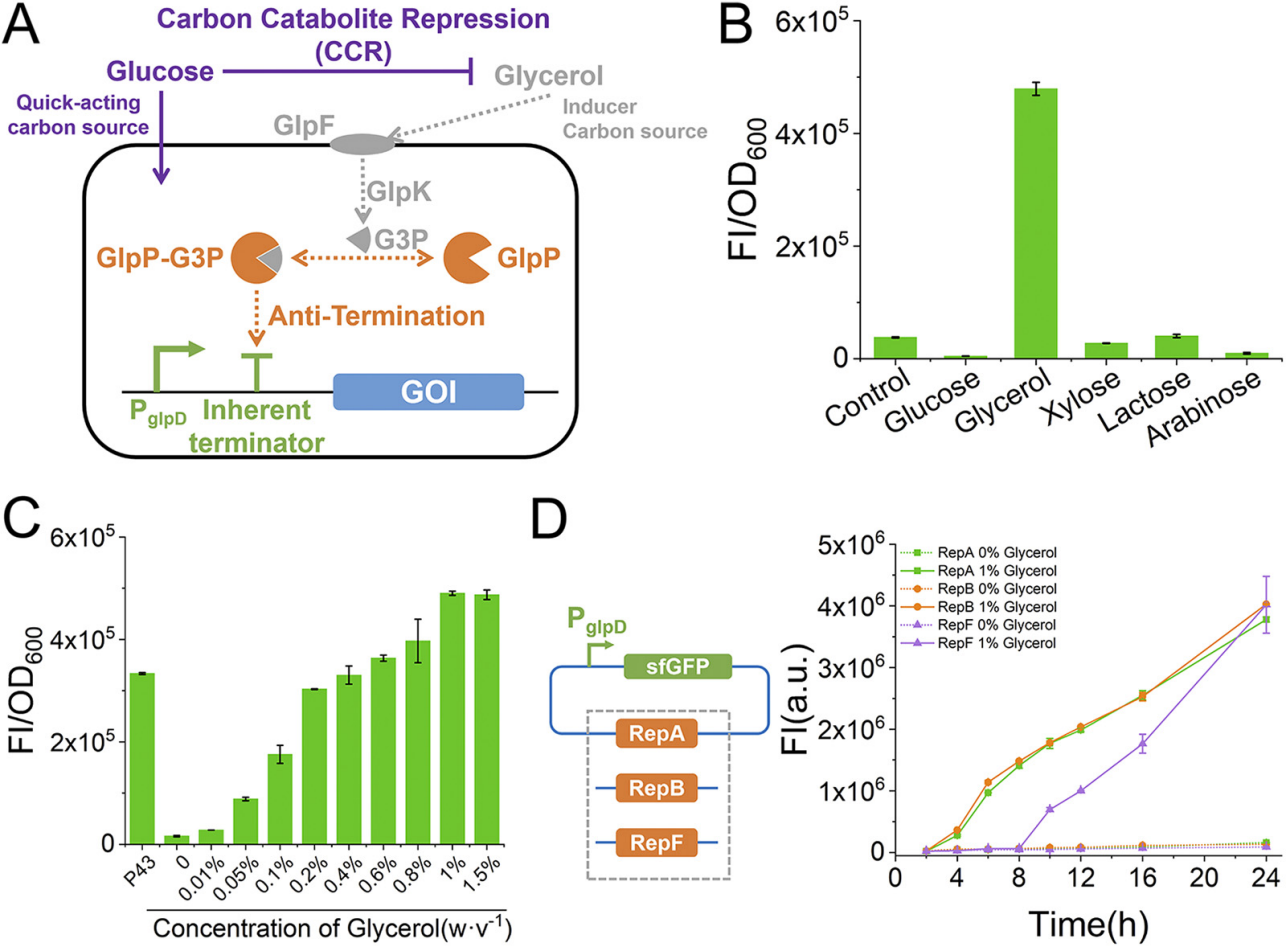
Design and construction of the GIES. (A) Schematic diagram of the GIES. (B) Specificity of the inducer in the GIES. The final concentration of each carbon source was 1% (wt/vol). Control means cell culture in LB medium. The fluorescence intensity (FI) and OD_600_ were determined after 24 h of cell culture. (C) Dose-dependent induction of sfGFP in the GIES by glycerol. The strong constitutive promoter P43 was employed as the control to determine the strength of P_glpD_. The fluorescence intensity and OD_600_ were determined after 24 h of cell culture. (D) Determination of the robustness of the GIES in different genetic environments.

A GIES that enables autoinducible expression of a GOI was proposed based on the theoretical B. subtilis sequential utilization of glucose and glycerol as carbon sources, which is regulated by CCR (Fig. 1A). In this system, the antiterminator protein GlpP binds the intermediate G3P during glycerol metabolism and further induces expression of the GOI by disrupting the inherent terminator of the GlpP-regulated promoter. In the presence of both glucose and glycerol, CCR guides B. subtilis to preferentially utilize glucose, and glycerol is consumed only when glucose is depleted. In this case, at the initiation of the fermentation process, glucose acts as a fast-acting carbon source to enable rapid biomass accumulation; when the glucose is used up, glycerol serves as both the second carbon source and the inducer of the GOI expression.

GlpP is known to specifically activate three promoters, namely, P_glpD_, P_glpT_, and P_glpFK_. The three promoters have similar structures, including the core region of the −35/−10 box, a GlpP binding site, and an inherent terminator located in the 5′ UTR (32, 33) (see Fig. S1 in the supplemental material). In addition, P_glpFK_ is negatively regulated by the catabolite control protein CcpA (27). First, the three promoters, consisting of 200-bp sequences upstream of their respective genes, were separately cloned, and their performance was verified by monitoring the expression of the reporter superfolder green fluorescent protein (sfGFP). As shown in Fig. S2 in the supplemental material, P_glpT_ showed the lowest activity induced by glycerol. Although P_glpFK_ showed high activity upon glycerol induction, its background activity was also high, resulting in a low induction ratio of 2.6-fold. In comparison, P_glpD_ showed a high induction ratio of 30.7-fold in the presence of 1% glycerol, resulting from its high induction and low background activity. Equally important, the activity of P_glpD_ was specifically induced by glycerol and was not affected by other common carbon sources. (Fig. 1B). Therefore, P_glpD_ was the ideal promoter for the construction of the GIES.

To verify the feasibility of the GIES, we constructed the recombinant strain BS-BPglpD-sfGFP, in which P_glpD_ controlled expression of the reporter sfGFP and RepB enabled plasmid replication. The BS-BPglpD-sfGFP strain was cultured in medium containing various concentrations of glycerol, and sfGFP expression exhibited a glycerol dose-dependent profile. As shown in Fig. 1C, sfGFP fluorescence intensity gradually increased as the concentration of glycerol in the medium increased (Fig. 1C; also see Fig. S3). Maximum expression of sfGFP was achieved when the concentration of glycerol reached 1% (wt/vol). Moreover, the expression driven by P_glpD_ was significantly higher than that noted with the strong constitutive promoter P43, which accounted for the high yield of protein obtained with the GIES. We also constructed alternative GIESs with vectors that utilize replication proteins RepA or RepF in place of RepB (Fig. 1D). In all three GIESs tested, sfGFP expression profiles were similar, that is, sfGFP was not expressed in the absence of glycerol but expression was efficiently induced by 1% glycerol (Fig. 1D). This result demonstrated the robustness of the GIES in different environments, which is crucial for future applications.

### Achievement of an autoinducible GIES through CCR-regulated carbon source switching.

When we proposed the construction of the GIES, we envisioned that, due to CCR-controlled regulation of gene expression, B. subtilis would preferentially utilize glucose before glycerol in a medium containing both glucose and glycerol. In this case, the GIES regulated the expression of the GOI in an autoinducible manner in which the induction of gene expression is coupled with the switch in carbon source from glucose to glycerol. To validate this assumption, the sfGFP expression profile of the BS-BPglpD-sfGFP construct in glucose and glycerol mixed media was characterized.

**FIG 2:**
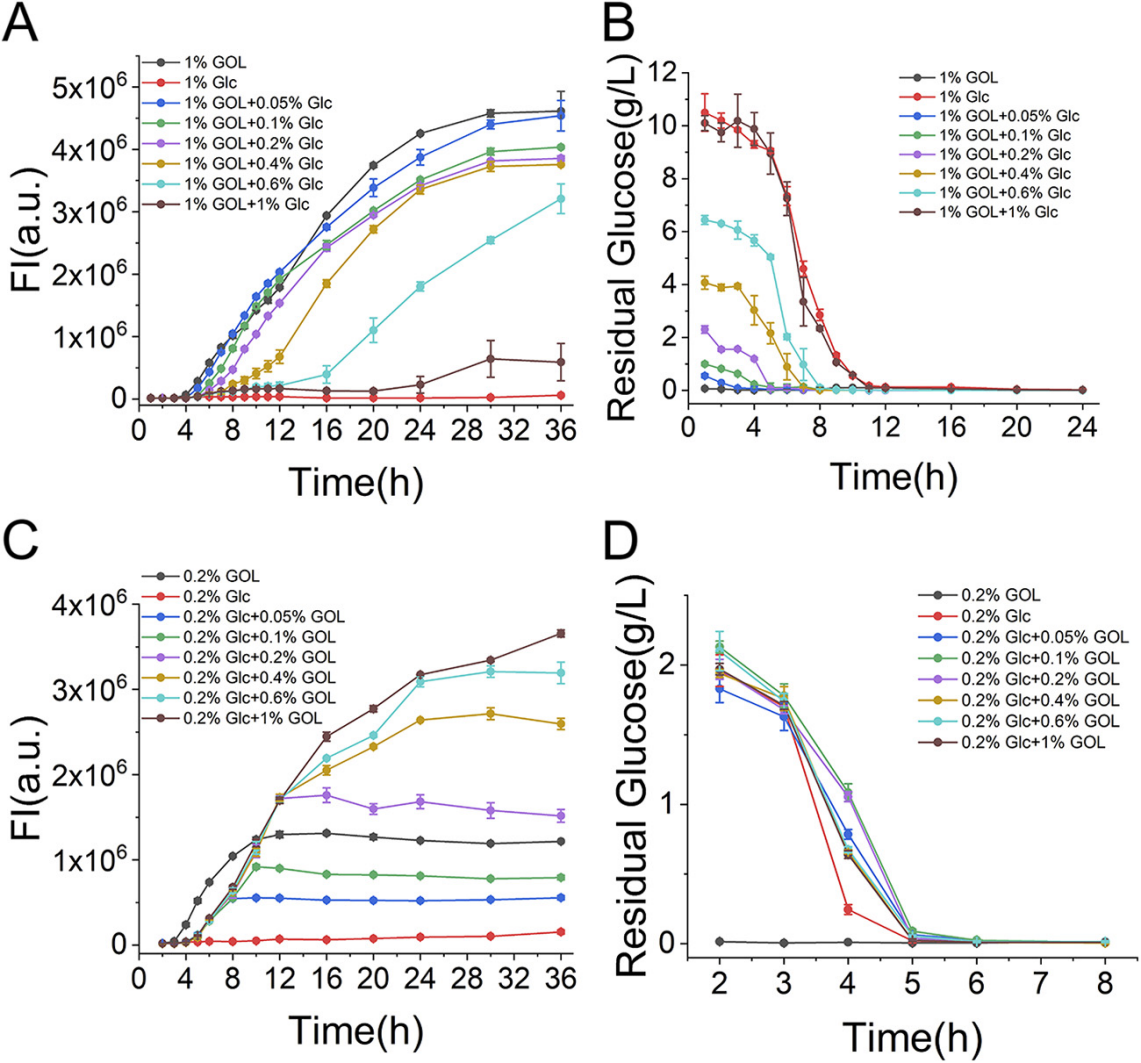
Characterization of the autoinducible GIES. (A) Profile of sfGFP expression with 1% (wt/vol) glycerol and various concentrations of glucose. The sfGFP expression with 1% glucose was set as the noninduced control and that with 1% glycerol as the control of constitutive expression. (B) Glucose consumption during cell growth with 1% (wt/vol) glycerol and various concentrations of glucose. (C) Profile of sfGFP expression with 0.2% (wt/vol) glucose and various concentrations of glycerol. The sfGFP expression with 0.2% glucose was set as the noninduced control and that with 0.2% glycerol as the control of constitutive expression. (D) Glucose consumption during cell growth with 0.2% (wt/vol) glucose and various concentrations of glycerol. FI, fluorescence intensity; GOL, glycerol; Glc, glucose.

When BS-BPglpD-sfGFP was cultured in medium with a fixed glycerol concentration (1%) but different concentrations of glucose (from 0.05% to 1% [wt/vol]), sfGFP appeared to be expressed in an autoinducible manner. Moreover, sfGFP induction exhibited a glucose dose-dependent delay, meaning that, when the glucose concentration was higher, the delay in the expression of sfGFP was greater (Fig. 2A). In addition, the onset of sfGFP induction and that of glucose depletion were strongly coupled, demonstrating the tight regulation of the autoinducible GIES (Fig. 2B). Notably, the final level of sfGFP yield was highest in the control group with 1% glycerol, and the delayed induction resulted in lower sfGFP expression levels. sfGFP is a heterologous protein that does not impose significant pressure on the host, and this autoinducible expression system did not exhibit any substantial advantage over constitutive expression. Further, we characterized the sfGFP expression profile when the clone was cultured in a medium with a constant glucose concentration (0.2% [wt/vol]) but gradient concentrations of glycerol (from 0.05% to 1% [wt/vol]). As shown in Fig. 2C, irrespective of the glycerol concentration, sfGFP induction began at similar times (about 5 h). This was consistent with the fact that glucose was almost depleted at 5 h (Fig. 2D). However, expression levels of sfGFP were noticeably governed by the glycerol concentration.

Overall, the autoinducible GIES was able to efficiently express the GOI as it automatically switched between glucose and glycerol regulated by CCR. In this system, glucose regulated the timing of induction, and the strength of the GOI expression was regulated by both glucose and glycerol. Therefore, it is reasonable to think that fine-tuning of GOI expression in the GIES can be achieved with a suitable ratio of glucose to glycerol. In addition, the noninduced sfGFP expression in the control group with only glucose demonstrated that the GOI expression in the GIES was specifically induced by glycerol and not carbon starvation (Fig. 2A and C).

### Further improvement of the GIES by overexpressing GlpP.

In the GIES, the process of GlpP disrupting the inherent terminator was vital for the activation of P_glpD_. To validate this, the GIES was constructed in the *glpP*-deficient strain B. subtilis (Δ*glpP*). In this system, sfGFP expression cannot be triggered by the GIES (see Fig. S4), which indicates that GlpP is the specific mediator for the activation of P_glpD_ by glycerol. Based on this, we speculated that the level of GlpP directly affected the GOI expression regulated by the GIES. Accordingly, an extra GlpP expression cassette was introduced to construct the strengthened GIES (SGIES) (see Fig. S6). The strains SGIES-sfGFP and SGIES-AspA, which express sfGFP and the E. coli aspartase AspA, respectively, were constructed to verify the higher yield of heterologous proteins with the SGIES.

**FIG 3:**
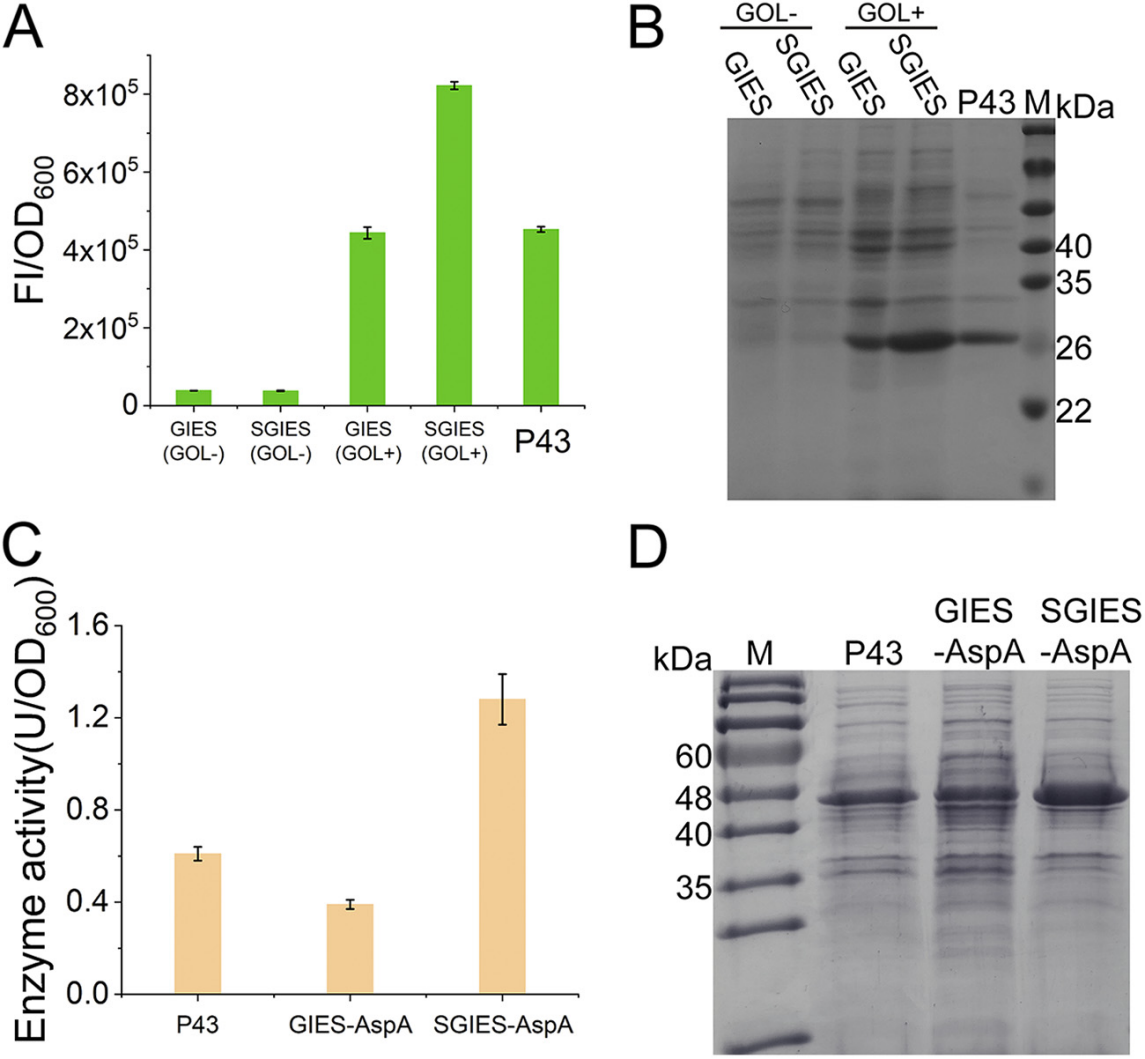
Verification of improved GOI expression with the SGIES. (A) Determination of sfGFP expression strength in the GIES and SGIES. GOL−, no glycerol added; GOL+, 0.5% (wt/vol) glycerol added; FI, fluorescence intensity. (B) SDS-PAGE analysis of sfGFP expression in the GIES and SGIES. (C) Overexpression of AspA in the SGIES. The cells expressing AspA by P43 were cultured in LB medium, and the cells expressing AspA in the GIES and SGIES were cultured in LB medium with 0.5% (wt/vol) glycerol. (D) SDS-PAGE analysis of AspA expression. M, marker.

In the SGIES-sfGFP strain with a higher GlpP level, sfGFP expression was significantly enhanced at a moderate glycerol concentration (0.5% [wt/vol]), with the expression level being about 2-fold greater than that in the GIES (Fig. 3A). Interestingly, in the absence of glycerol, a higher level of GlpP did not result in significantly higher background expression (Fig. 3A and B). This result further indicated the specificity and rigor of GlpP regulation. A similar profile was observed for the expression of AspA. The AspA expression level driven by GIES with 0.5% glycerol was even lower than that driven by P43. When the SGIES was applied, AspA expression was considerably strengthened (Fig. 3C and D).

For the development of an inducible expression system, the regulator's expression level is often critical. For example, when the lactose-inducible *lac* promoter was introduced on a high-copy-number plasmid, a high level of LacI was necessary (34). When the RcnR binding site was used to regulate the constitutive promoter on the plasmid, chromosomal expression of RcnR was insufficient to suppress promoter activity (6). For the synthetic hybrid promoter P_XYL_ in Saccharomyces cerevisiae, which was repressed by the repressor XylR, the lack of XylR resulted in constitutive expression of the GOI (35). Here, a similar result was obtained in the antiterminator protein-regulated expression system.

### Highly efficient expression of NK by the autoinducible SGIES.

NK, a serine protease with high fibrin-degrading activity, has great prospects for application in the health care and medical fields (36). However, its high protein degradation activity exerts considerable stress on the host. In addition, poor thermostability and strong autodegradation make it difficult for the protease to exist stably after secretion into the fermentation liquid. Thus, the autoinducible SGIES is well suited to achieve a fine balance between cell growth and NK expression for optimal biomass and highest NK yield, which is difficult to realize using constitutive expression systems.

**FIG 4:**
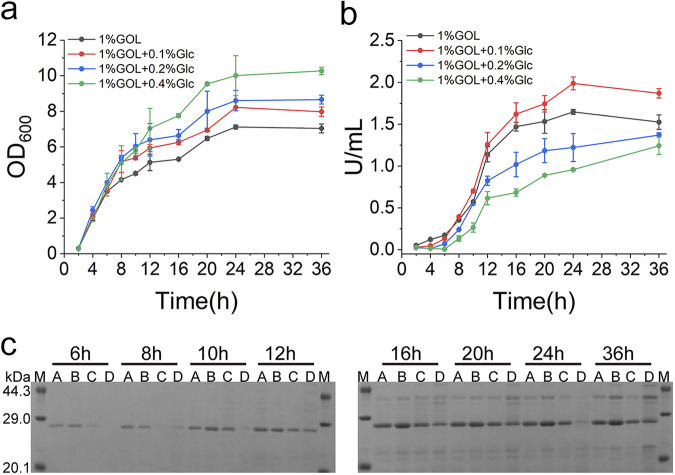
Characterization of NK expression in the autoinducible SGIES. (a) Growth of cells expressing NK in the autoinducible SGIES. (b) Expression of NK in the autoinducible SGIES. (c) SDS-PAGE analysis of NK expression in the autoinducible SGIES. A, 1% glycerol; B, 1% glycerol and 0.1% glucose; C, 1% glycerol and 0.2% glucose; D, 1% glycerol and 0.4% glucose. M, marker.

Thus, the autoinducible SGIES was applied in the protease-deficient strain WB800 to obtain a high yield of NK (WB-SGIES-NK). Different concentrations of glucose (0.1%, 0.2%, and 0.4%) were used as the preferred carbon source and to inhibit NK expression during the cell growth phase. One percent glycerol was used as the second carbon source as well as the inducer of NK expression during the production phase. When cells were cultured in a medium containing only glycerol, NK was expressed at the beginning and cell growth was significantly inhibited, most likely due to the stress placed on the host (Fig. 4a and b). With the addition of glucose, cell growth was significantly accelerated and a greater biomass was achieved during fermentation (Fig. 4a). The production of NK was progressively delayed with increasing glucose concentrations (Fig. 4b). As expected, delaying the induction reduced the final NK yield, which corresponded to sfGFP expression driven by the autoinducible SGIES. NK expression under different autoinducible conditions was analyzed by SDS-PAGE and demonstrated that proper decoupling of cell growth and gene expression facilitated NK production (Fig. 4c). In general, the addition of 0.1% glucose was most favorable for the efficient production of NK.

As reported previously, a quorum-sensing-based autoinducible expression system was constructed for efficient expression of NK in Bacillus subtilis (37). Here, we further clarified the advantages of the autoinducible approach for high yields of NK. In fact, this autoinducible approach should facilitate the production of most products that cause stress to the host, such as membrane proteins and antimicrobial peptides. Therefore, the GIES is expected to have a wider range of applications.

### Scale-up production of NK by the SGIES in a 5-L fermenter.

**FIG 5:**
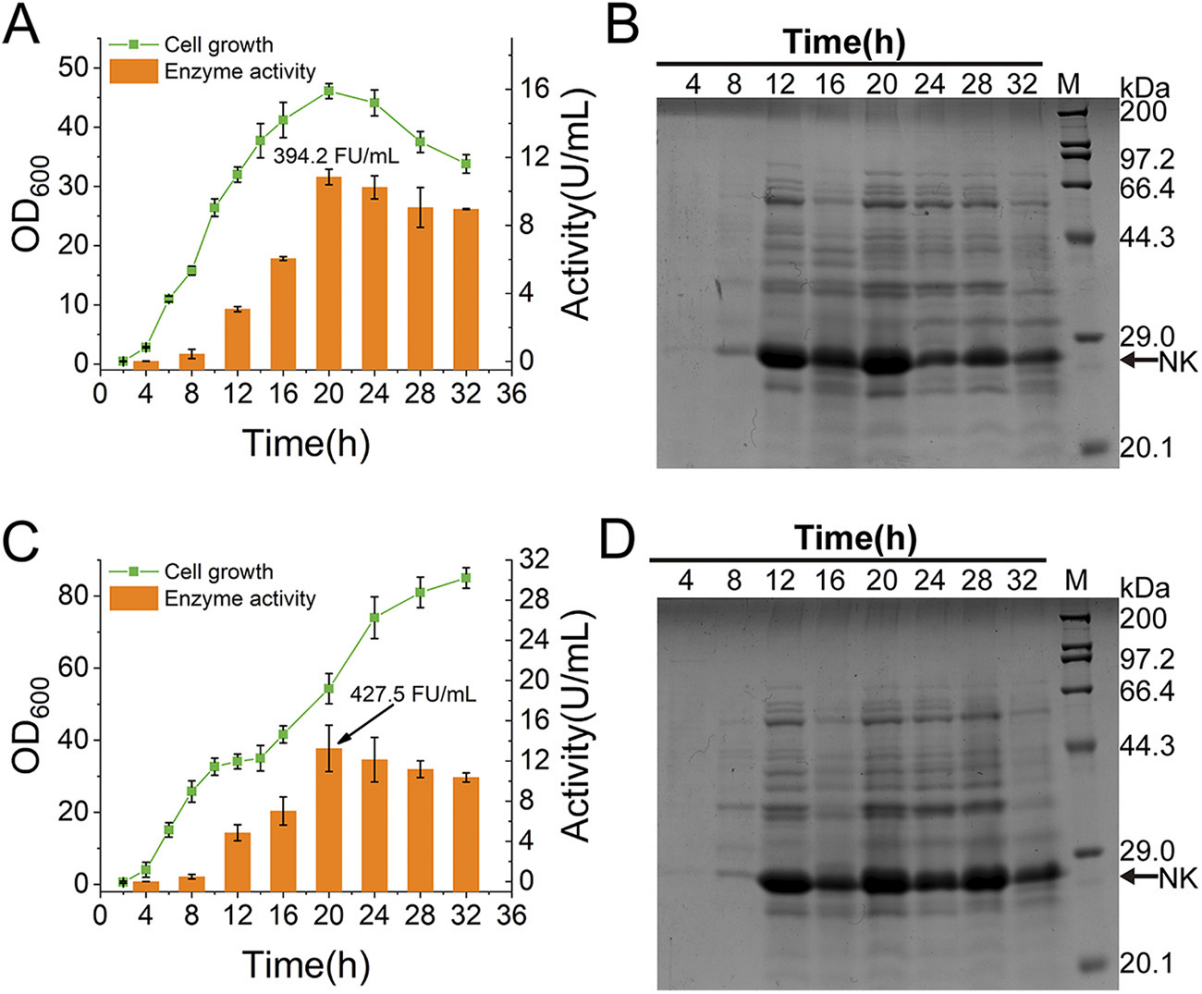
Scale-up production of NK in a 5-L fermenter. (A) Characterization of cell growth and NK production by batch fermentation. (B) SDS-PAGE analysis of NK production by batch fermentation. (C) Characterization of cell growth and NK production by fed-batch fermentation. (D) SDS-PAGE analysis of NK production by fed-batch fermentation. The fibrinolytic activity of NK in supernatant is labeled.

The WB-SGIES-NK strain was employed to validate the expression of NK by the autoinducible SGIES when production was scaled-up in a 5-L fermenter under batch or fed-batch fermentation. For batch fermentation, 0.5% (wt/vol) glucose served as the quick-acting carbon source for rapid cell growth, and 5% (wt/vol) glycerol served as the secondary carbon source and inducer of NK expression during the production phase. During batch fermentation, the cells started to grow rapidly from 2 h postinoculation, with a short lag phase due to the preferential utilization of glucose (Fig. 5A). After the depletion of glucose at 6 h, the cells maintained the high growth rate while NK was expressed at high levels (Fig. 5A). Both cell growth (optical density at 600 nm [OD_600_]) and enzyme activity peaked at 20 h, at an OD_600_ of 46.1 ± 1.3 and an enzyme activity of 10.9 ± 0.5 U/mL (394.2 fibrinolytic units [FU]/mL of fibrin degradation activity). After this, the OD_600_ and enzyme activity progressively decreased until the end of fermentation.

For the feed-batch fermentation, the starter medium contained 0.5% (wt/vol) glucose and 2% (wt/vol) glycerol. The profiles of cell growth and NK expression in the early stage were similar to those during the batch fermentation (Fig. 5C). However, with continuous supplementation of glycerol and nitrogen sources from 12 h onwards, the cells entered a second rapid growth phase until the end of the fermentation, with the OD_600_ reaching 85.0 ± 2.8 (Fig. 5C). Benefiting from the higher biomass, the NK yield from fed-batch fermentation was also higher than that from batch fermentation, and an enzyme activity of 13.3 ± 2.3 U/mL (427.5 FU/mL of fibrin degradation activity) was obtained at 20 h (Fig. 5C). Although enzyme activity progressively decreased after 20 h, most likely as a result of NK instability, activity was still higher than that in batch fermentation.

Microbial production is an ideal way to obtain NK at low cost, and many attempts have been made (38). In this study, the GIES was applied to achieve a high level of secretory expression of NK. Although the level of NK expression in the GIES was lower than that recently reported for NK expression using a stronger tandem promoter, it was already higher than levels noted with other expression systems (39). It is thought that, with modification, the GIES can be further improved.

### Exploration of extending the application of P_glpD_ in synthetic biology.

**FIG 6:**
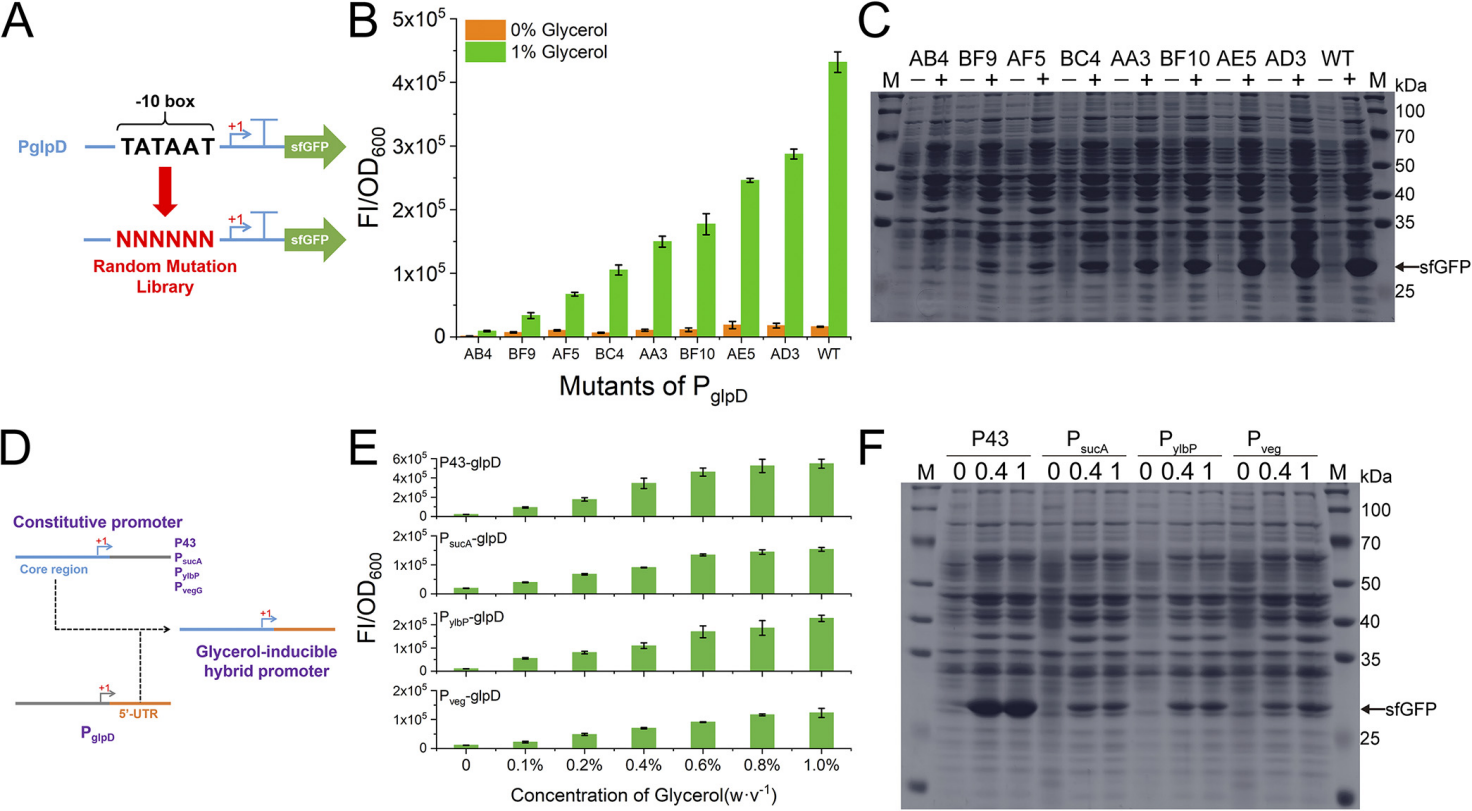
Extending the application of P_glpD_ in synthetic biology. (A) Schematic diagram of the construction of a random mutation library targeting the −10 box of P_glpD_. (B) Characterization of the P_glpD_ mutants screened from the random mutation library. FI, fluorescence intensity. (C) SDS-PAGE analysis of the sfGFP expression driven by P_glpD_ mutants. −, without glycerol addition; +, sfGFP expression induced by 1% (wt/vol) glycerol. (D) Schematic diagram of the construction of hybrid promoters. (E) Characterization of the sfGFP expression driven by hybrid promoters with various concentrations of glycerol. (F) SDS-PAGE analysis of the sfGFP expression driven by hybrid promoters with 0%, 0.4% and 1% glycerol. N stands for 4 bases (A, T, C, and G) with the same probability. +1, TSS.

Fine-tuning of gene expression is crucial in synthetic biology and metabolic engineering (40, 41). Promoters are the core biological parts for fine-tuning gene expression because they regulate gene transcription at the most fundamental level (42). Numerous studies have shown that promoter mutants with different activities can be obtained by diversifying the sequences of the core promoter region (43–45). Here, we targeted the −10 box of P_glpD_, which is responsible for specific binding of RNA polymerases, and constructed a random mutation library by introducing N (Fig. 6A). Using sfGFP as a reporter, 180 single colonies from more than 1,000 transformants were preliminarily screened in a 96-well plate. These cells expressed sfGFP at different levels under 1% glycerol induction; most of the levels were lower than those noted with the wild-type (WT) P_glpD_, and a few were slightly higher than WT levels (see Fig. S5). After further validation in test tubes, eight mutants with gradient activities were finally obtained, and they all showed lower activities than the WT strain, although AD3 showed slightly higher activity than the WT strain in 96-well plate screening (Fig. 6B). Importantly, all of these mutants retained strict glycerol-inducible properties, with extremely low levels of sfGFP expression in the absence of glycerol and apparent sfGFP induction by glycerol. In addition, the sequences of the −10 box were closely related to their activities (see Table S3). The −10 box sequence of P_glpD_ is TATAAT, which is also the consensus sequence recognized by σ^A^ in Bacillus subtilis (46), and thus the WT strain showed high activity. For P_glpD_ mutants, the strains with greater differences from the consensus sequence showed lower activities. The fluorescence detection was confirmed by the SDS-PAGE analysis (Fig. 6C).

Essentially, the interaction between the antiterminator protein GlpP and the inherent terminator is the molecular basis for the glycerol-inducible regulation of P_glpD_. Theoretically, the inherent terminator is expected to be developed as a portable block that, in combination, imparts glycerol-regulated properties to other biological parts. For instance, combining it with regular constitutive promoters ought to generate novel hybrid glycerol-inducible promoters (Fig. 6D). To validate this assumption, we constructed hybrid promoters with the 5′ UTR of P_glpD_ downstream of the core region of four typical constitutive promoters (P43, P_sucA_, P_ylbP_, and P_veg_) that have been widely used for promoter engineering (5, 45, 47) (see Table S4). Here, the core region included the −35 box, the −10 box, and the transcription start site (TSS), and 10 bp of 3′ flanking sequence of the TSS was retained to ensure that transcription started smoothly. In this way, four hybrid promoters (P43-glpD, P_sucA_-glpD, P_ylbP_-glpD, and P_veg_-glpD) were constructed and characterized in test tubes using sfGFP as the reporter. As expected, these hybrid promoters exhibited glycerol-inducible properties. sfGFP was barely expressed in the absence of glycerol, whereas sfGFP expression was significantly induced with increasing glycerol concentrations (Fig. 6E). Among these hybrid promoters, P43-glpD showed the highest activity; P_sucA_-glpD and P_ylbP_-glpD were similar in activity and significantly lower than P43-glpD; while P_veg_-glpD had the lowest activity. Notably, the glycerol dose-dependent profiles of the activities of the hybrid promoters were similar to that of P_glpD_, and their activities could be almost completely induced by 1% glycerol (Fig. 1C and 6E). This result demonstrated that the regulatory effects of GlpP and the inherent terminator are similar and stable, either in P_glpD_ or in the hybrid promoters. The glycerol-inducible sfGFP expression driven by the hybrid promoters was confirmed by the SDS-PAGE analysis (Fig. 6F).

The evolution of the −10 box of P_glpD_ yielded mutants with different activities, providing more choices for diverse applications. It also indicated relative independence between RNA polymerase-dependent transcription initiation and regulation of the inherent terminator in P_glpD_. It was demonstrated previously that engineering the core region of transcription factor-regulated promoters can fine-tune their activity, as well as their dynamic range (48). Here, we demonstrated that the engineering of promoters regulated by antiterminator proteins was also amenable to this principle. Further, we successfully conferred glycerol-inducible regulation to the constitutive promoters by combining the 5′ UTR of P_glpD_. The construction of hybrid inducible promoters based on transcription factor regulation has been reported in prokaryotic and eukaryotic cells (6, 49, 50), and we demonstrated that similar combination was also feasible for the antiterminator regulator. This result suggested that the pair of inherent terminator and antiterminator protein is expected to be developed as a portable biological tool for a variety of purposes in synthetic biology, such as the construction of complex logic gates and the regulation of gene expression in polycistron.

### Conclusion.

In this study, a GIES was constructed in B. subtilis using promoter P_glpD_. The GIES was capable of expressing the GOI at high levels in a variety of genetic environments. In addition, based on the sequential utilization of glucose and glycerol by B. subtilis, GIES was able to express the GOI in an autoinducible manner in a medium containing both glucose and glycerol. After the depletion of glucose, glycerol was used as an alternative carbon source and induced the expression of GOI. The GOI expression profiles with various concentrations of glucose and glycerol showed that the dose of glycerol regulated the GOI expression level and the dose of glucose affected both the induction timing and the GOI expression level. The GIES was successfully applied to a high yield of NK and also showed that the autoinducible approach facilitated the synthesis of products that cause stress to the host.

In addition, we explored the expanding applications of P_glpD_ in synthetic biology. Through the evolution of the −10 box, mutants of P_glpD_ with gradient activities were obtained. Furthermore, hybrid glycerol-inducible promoters were constructed by combining the constitutive promoters and the 5′ UTR of P_glpD_. This work not only provided additional tools for fine-tuning gene expression but also demonstrated that antiterminator protein-regulated promoters shared similar engineering strategies with constitutive promoters and classic transcription factor-regulated promoters.

Overall, this study developed an efficient GIES to synthesize high-value-added products with inexpensive glycerol. More importantly, it demonstrated clearly the great potential of using an antiterminator protein as the regulator for a variety of purposes in synthetic biology.

## MATERIALS AND METHODS

### Strains, plasmids, and culture conditions.

E. coli JM109 was employed for plasmid construction and propagation. B. subtilis 168 and B. subtilis WB800 were employed for the expression of intracellular and secreted proteins, respectively. The genes of P_glpD_, P_glpT_, P_glpFK_, and *glpP* were amplified from the extracted genome of B. subtilis 168. The genes of RepA and RepF were cloned from the plasmids pHT01 and pCasSA (51), respectively. Luria-Bertani (LB) medium (10 g/L tryptone, 5 g/L yeast extract, 10 g/L NaCl [pH 7.0]) was used for cell culture and the expression of sfGFP and AspA. Terrific broth (TB) medium (12 g/L tryptone, 24 g/L yeast extract, 4 g/L NaCl, 17 mM KH_2_PO_4_, 72 mM K_2_HPO_4_) was used for the high-level secretory expression of NK. For preparation of solid media, 1.5% agar was added. Unless otherwise noted, cells were normally cultured at 37°C; for the recombinant strain harboring temperature-sensitive plasmids containing RepF, cells were cultured at 30°C. The concentrations of antibiotics for selection and growth that were added to the media were 100 μg/mL ampicillin and 5 μg/mL kanamycin. All of the strains and plasmids used in this study are listed in Table S1 in the supplemental material. The plasmid maps are shown in Fig. S6.

### Genetic manipulation.

In this study, all plasmids were constructed by the seamless cloning technique, based on Gibson assembly, reported previously (52). The high-fidelity PrimeSTAR Max DNA polymerase (TaKaRa Bio Inc., Japan) was used for the amplification of DNA fragments required for plasmid construction. The PCR products were treated with DpnI (TaKaRa Bio Inc.) and then cleaned up with a DNA clean-up kit (CWBIO, China) to remove the templates. The ABclonal MultiF seamless assembly mix (ABclonal Technology Co. Ltd., China) was used for the seamless ligation of purified fragments. The reaction product was directly transformed into E. coli JM109 competent cells. The constructed plasmids were then extracted and transformed into B. subtilis 168 or WB800 cells. The primers used in this study are listed in Table S2 in the supplemental material. For the *in vitro* assembly of amplified fragments, 15- to 3-bp-long homologous sequences were designed in the primers.

### Fluorescence assays.

sfGFP served as the reporter to characterize the GIES. The single clone was inoculated into a test tube containing 5 mL LB medium. After overnight culture at 37°C with shaking at 200 rpm, 1 mL of seed liquid was transferred into 250-mL shake flasks containing 50 mL of LB medium and further incubated at 37°C with shaking at 200 rpm. As needed, appropriate concentrations of glucose and glycerol were added to regulate the expression of sfGFP. The cultured cells were harvested by centrifugation at 12,000 rpm for 3 min and then washed three times with phosphate-buffered saline (PBS) (8 g/L NaCl, 0.2 g/L KCl, 1.44 g/L Na_2_HPO_4_, 0.24 g/L KH_2_PO_4_ [pH 7.4]). After being resuspended in PBS, 200 μL of cells was transferred into 96-well black-walled plates. The OD_600_ and fluorescence intensity of sfGFP were determined with a Synergy H4 multimode microplate reader (excitation at 485 nm and emission at 528 nm).

### AspA enzymatic activity assays.

The cultured ApsA-expressing cells were harvested by centrifugation at 12,000 rpm for 3 min. The pellets were washed three times with PBS and then resuspended in 50 mM Tris-HCl (pH 8.0) at an OD_600_ of 3.0. The enzyme-catalyzed reaction solution was 1 mL, including 500 μL substrate of 200 mM fumaric acid (dissolved in ammonium hydroxide solution, with the pH adjusted to 8.0 with HCl) and 500 μL cell suspension. The reaction was carried out in a metal bath at 37°C and terminated by thermal inactivation at 100°C for 10 min. The product l-aspartate was derivatized with phenyl isothiocyanate, and the concentration was determined using a C_18_ column [Diamonsil C_18_(2), 5 μm, 250 by 4.6 mm; DIKMA, China] by high-performance liquid chromatography (HPLC) as described previously (53). One unit of AspA activity was defined as the amount of enzyme needed for the production of 1 μmol of l-aspartate per minute.

### NK enzymatic activity assays.

NK activity was determined with the substrate *N*-succinyl-l-Ala-l-Ala-l-Pro-l-Phe-*p*-nitroanilide (suc-AAPF-pNA) (Sigma, USA). The cell cultures were centrifuged at 12,000 rpm for 3 min, and the supernatant was used to determine the activity of secretory NK; 200 μL of the reaction system was added to a 96-well plate, including 40 μL of enzyme solution, 120 μL of buffer (100 mM Tris-HCl, 0.1 mM CaCl_2_ [pH 8.0]), and 40 μL of 20 mM suc-AAPF-pNA. The reaction was carried out at 25°C, and the variation of OD_405_ was measured with a Synergy H4 multimode microplate reader. One unit was defined as the amount of enzyme needed for the production of 1 μmol of the product *p*-nitroaniline per minute.

The fibrin degradation activity of NK was determined according the method described by the Japan NattoKinase Association (http://j-nattokinase.org/en/jnka_nattou_01.html), for comparison with other studies on NK production. In brief, 700 μL of fibrinogen solution (0.72% [wt/vol]), 200 μL of Tris-HCl (100 mM [pH 8.0]), and 50 μL of thrombin (20 U/mL) were mixed and incubated at 37°C for 10 min. Then, 100 μL of appropriately diluted fermentation supernatant was added, and the reaction was carried out at 37°C for 60 min. The reaction was terminated by the addition of 1 mL of 200 mM trichloroacetic acid. After centrifugation at 12,000 rpm for 15 min, the OD_275_ of the supernatant was measured with a UV-1800/PC spectrophotometer (MAPADA Instrument Co., Ltd.). One FU was defined as the amount of enzyme required to increase OD_275_ by 0.01 per minute.

### SDS-PAGE.

In this study, the protein expression was visualized by SDS-PAGE analysis. For the intracellular protein assay, cells were pretreated with cell lysis solution (20 mM Tris-HCl, 0.2 mg/mL lysozyme [pH 8.0]). The 5× loading buffer containing phenylmethylsulfonyl fluoride was used to prepare the NK sample for SDS-PAGE.

### Fermentation conditions in the 5-L fermenter.

**(i) Batch fermentation.** A single colony was inoculated into a test tube containing 5 mL TB and incubated at 37°C at 200 rpm for 12 h. Then, 800 μL of culture was inoculated into a shake flask containing 40 mL of TB and was incubated at 37°C at 200 rpm for 12 h. All secondary seed liquid was inoculated into the fermenter containing 2 L of fermentation medium (12 g/L tryptone, 24 g/L yeast extract, 5 g/L glucose anhydrous, 50 g/L glycerol, 17 mM KH_2_PO_4_, 72 mM K_2_HPO_4_, 0.2 g/L CaCl_2_).

**(ii) Fed-batch fermentation.** The seed fluid was cultured in two stages, as in the batch fermentation. The 2 L of starting medium for fed-batch fermentation consists of 12 g/L tryptone, 24 g/L yeast extract, 5 g/L glucose anhydrous, 20 g/L glycerol, 17 mM KH_2_PO_4_, 72 mM K_2_HPO_4_, and 0.2 g/L CaCl_2_. After about 12 h, fed-batch cultivation was started by feeding the supplemented medium (50 g/L glycerol, 12 g/L tryptone, 24 g/L yeast extract) at a rate of 0.4 to 1.0 mL/min. During the fermentation process, the culture temperature was kept at 37°C, the pH was kept at 7.0 with NH_4_OH and 20% (vol/vol) H_3_PO_4_, the airflow rate was set to 7 L/min, and the agitation speed was automatically adjusted between 200 and 900 rpm to ensure that the dissolved oxygen concentration did not fall below 30%.

**Construction and screening of the random mutation library of the −10 box in P_glpD_.** Randomized mutations were introduced into the plasmid pBPglpD-sfGFP by degenerate oligonucleotides N, replacing the −10 box of the WT P_glpD_ (5′-TATAAT-3′). The purified PCR products were seamlessly cloned and transformed into E. coli JM109 to construct plasmids containing a P_glpD_ mutant library. Then, the random mutation library was transformed into B. subtilis 168, and the transformed cells were plated onto an LB agar plate with 1% (wt/vol) glycerol. Upon observation under blue light, 180 single colonies showing different fluorescence intensities were picked out from over 1,000 transformants and cultured on 96-well plates containing 600 μL LB medium with 1% (wt/vol) glycerol at 37°C at 300 rpm for 24 h. Mutants with different sfGFP expression levels were further verified in test tubes and sequenced.

### Data availability.

All data included in this study are available upon request by contact with the corresponding author.

## References

[1] Jung SW, Yeom J, Park JS, Yoo SM. 2021. Recent advances in tuning the expression and regulation of genes for constructing microbial cell factories. Biotechnol Adv 50:107767. doi:10.1016/j.biotechadv.2021.107767. 33974979

[2] Jin LQ, Jin WR, Ma ZC, Shen Q, Cai X, Liu ZQ, Zheng YG. 2019. Promoter engineering strategies for the overproduction of valuable metabolites in microbes. Appl Microbiol Biotechnol 103:8725–8736. doi:10.1007/s00253-019-10172-y. 31630238

[3] Fitz E, Wanka F, Seiboth B. 2018. The promoter toolbox for recombinant gene expression in *Trichoderma reesei*. Front Bioeng Biotechnol 6:135. doi:10.3389/fbioe.2018.00135. 30364340 PMC6193071

[4] de Grahl I, Rout SS, Maple-Grødem J, Reumann S. 2020. Development of a constitutive and an auto-inducible high-yield expression system for recombinant protein production in the microalga *Nannochloropsis oceanica*. Appl Microbiol Biotechnol 104:8747–8760. doi:10.1007/s00253-020-10789-4. 32902683 PMC7502441

[5] Han L, Chen Q, Lin Q, Cheng J, Zhou L, Liu Z, Guo J, Zhang L, Cui W, Zhou Z. 2020. Realization of robust and precise regulation of gene expression by multiple sigma recognizable artificial promoters. Front Bioeng Biotechnol 8:92. doi:10.3389/fbioe.2020.00092. 32140461 PMC7042180

[6] Han L, Cui W, Lin Q, Chen Q, Suo F, Ma K, Wang Y, Hao W, Cheng Z, Zhou Z. 2020. Efficient overproduction of active nitrile hydratase by coupling expression induction and enzyme maturation via programming a controllable cobalt-responsive gene circuit. Front Bioeng Biotechnol 8:193. doi:10.3389/fbioe.2020.00193. 32266230 PMC7105576

[7] Shen X, Wang J, Li C, Yuan Q, Yan Y. 2019. Dynamic gene expression engineering as a tool in pathway engineering. Curr Opin Biotechnol 59:122–129. doi:10.1016/j.copbio.2019.03.019. 31063878

[8] Zhou P, Fang X, Xu N, Yao Z, Xie W, Ye L. 2021. Development of a highly efficient copper-inducible GAL regulation system (CuIGR) in *Saccharomyces cerevisiae*. ACS Synth Biol 10:3435–3444. doi:10.1021/acssynbio.1c00378. 34874147

[9] Gupta A, Reizman IM, Reisch CR, Prather KL. 2017. Dynamic regulation of metabolic flux in engineered bacteria using a pathway-independent quorum-sensing circuit. Nat Biotechnol 35:273–279. doi:10.1038/nbt.3796. 28191902 PMC5340623

[10] Waters CM, Bassler BL. 2005. Quorum sensing: cell-to-cell communication in bacteria. Annu Rev Cell Dev Biol 21:319–346. doi:10.1146/annurev.cellbio.21.012704.131001. 16212498

[11] Guan C, Cui W, Cheng J, Zhou L, Guo J, Hu X, Xiao G, Zhou Z. 2015. Construction and development of an auto-regulatory gene expression system in *Bacillus subtilis*. Microb Cell Fact 14:150. doi:10.1186/s12934-015-0341-2. 26392346 PMC4578258

[12] Studier FW. 2005. Protein production by auto-induction in high density shaking cultures. Protein Expr Purif 41:207–234. doi:10.1016/j.pep.2005.01.016. 15915565

[13] Ying Q, Zhang C, Guo F, Wang S, Bie X, Lu F, Lu Z. 2012. Secreted expression of a hyperthermophilic α-amylase gene from *Thermococcus* sp. HJ21 in *Bacillus subtilis*. J Mol Microbiol Biotechnol 22:392–398. doi:10.1159/000346215. 23486110

[14] Ming-Ming Y, Wei-Wei Z, Xi-Feng Z, Pei-Lin C. 2006. Construction and characterization of a novel maltose inducible expression vector in *Bacillus subtilis*. Biotechnol Lett 28:1713–1718. doi:10.1007/s10529-006-9146-z. 17001500

[15] Gasser B, Steiger MG, Mattanovich D. 2015. Methanol regulated yeast promoters: production vehicles and toolbox for synthetic biology. Microb Cell Fact 14:196. doi:10.1186/s12934-015-0387-1. 26627685 PMC4667464

[16] Martínez-Gómez K, Flores N, Castañeda HM, Martínez-Batallar G, Hernández-Chávez G, Ramírez OT, Gosset G, Encarnación S, Bolivar F. 2012. New insights into *Escherichia coli* metabolism: carbon scavenging, acetate metabolism and carbon recycling responses during growth on glycerol. Microb Cell Fact 11:46. doi:10.1186/1475-2859-11-46. 22513097 PMC3390287

[17] De Mey M, De Maeseneire S, Soetaert W, Vandamme E. 2007. Minimizing acetate formation in *E. coli* fermentations. J Ind Microbiol Biotechnol 34:689–700. doi:10.1007/s10295-007-0244-2. 17668256

[18] Wei L, Zhao J, Wang Y, Gao J, Du M, Zhang Y, Xu N, Du H, Ju J, Liu Q, Liu J. 2022. Engineering of *Corynebacterium glutamicum* for high-level γ-aminobutyric acid production from glycerol by dynamic metabolic control. Metab Eng 69:134–146. doi:10.1016/j.ymben.2021.11.010. 34856366

[19] Luo X, Ge X, Cui S, Li Y. 2016. Value-added processing of crude glycerol into chemicals and polymers. Bioresour Technol 215:144–154. doi:10.1016/j.biortech.2016.03.042. 27004448

[20] da Silva GP, Mack M, Contiero J. 2009. Glycerol: a promising and abundant carbon source for industrial microbiology. Biotechnol Adv 27:30–39. doi:10.1016/j.biotechadv.2008.07.006. 18775486

[21] Cui W, Han L, Suo F, Liu Z, Zhou L, Zhou Z. 2018. Exploitation of *Bacillus subtilis* as a robust workhorse for production of heterologous proteins and beyond. World J Microbiol Biotechnol 34:145. doi:10.1007/s11274-018-2531-7. 30203131

[22] Zhang K, Su L, Wu J. 2020. Recent advances in recombinant protein production by *Bacillus subtilis*. Annu Rev Food Sci Technol 11:295–318. doi:10.1146/annurev-food-032519-051750. 31905010

[23] Gu Y, Xu X, Wu Y, Niu T, Liu Y, Li J, Du G, Liu L. 2018. Advances and prospects of *Bacillus subtilis* cellular factories: from rational design to industrial applications. Metab Eng 50:109–121. doi:10.1016/j.ymben.2018.05.006. 29775652

[24] Nguyen QA, Schumann W. 2014. Use of IPTG-inducible promoters for anchoring recombinant proteins on the *Bacillus subtilis* spore surface. Protein Expr Purif 95:67–76. doi:10.1016/j.pep.2013.11.018. 24326192

[25] Yue J, Fu G, Zhang D, Wen J. 2017. A new maltose-inducible high-performance heterologous expression system in *Bacillus subtilis*. Biotechnol Lett 39:1237–1244. doi:10.1007/s10529-017-2357-7. 28527120

[26] Phan TT, Schumann W. 2007. Development of a glycine-inducible expression system for *Bacillus subtilis*. J Biotechnol 128:486–499. doi:10.1016/j.jbiotec.2006.12.007. 17208325

[27] Darbon E, Servant P, Poncet S, Deutscher J. 2002. Antitermination by GlpP, catabolite repression via CcpA and inducer exclusion triggered by P-GlpK dephosphorylation control *Bacillus subtilis glpFK* expression. Mol Microbiol 43:1039–1052. doi:10.1046/j.1365-2958.2002.02800.x. 11929549

[28] Holmberg C, Rutberg L. 1992. An inverted repeat preceding the *Bacillus subtilis glpD* gene is a conditional terminator of transcription. Mol Microbiol 6:2931–2938. doi:10.1111/j.1365-2958.1992.tb01752.x. 1479885

[29] Fujita Y. 2009. Carbon catabolite control of the metabolic network in *Bacillus subtilis*. Biosci Biotechnol Biochem 73:245–259. doi:10.1271/bbb.80479. 19202299

[30] Chen Q, Cui W, Zhou Z, Han L. 2021. Exploration of key residues and conformational change of anti-terminator protein GlpP for ligand and RNA binding. Proteins 89:623–631. doi:10.1002/prot.26045. 33455022

[31] Zhang XZ, Cui ZL, Hong Q, Li SP. 2005. High-level expression and secretion of methyl parathion hydrolase in *Bacillus subtilis* WB800. Appl Environ Microbiol 71:4101–4103. doi:10.1128/AEM.71.7.4101-4103.2005. 16000826 PMC1169017

[32] Glatz E, Nilsson RP, Rutberg L, Rutberg B. 1996. A dual role for the *Bacillus subtilis glpD* leader and the GlpP protein in the regulated expression of *glpD*: antitermination and control of mRNA stability. Mol Microbiol 19:319–328. doi:10.1046/j.1365-2958.1996.376903.x. 8825777

[33] Holmberg C, Rutberg B. 1991. Expression of the gene encoding glycerol-3-phosphate dehydrogenase (*glpD*) in *Bacillus subtilis* is controlled by antitermination. Mol Microbiol 5:2891–2900. doi:10.1111/j.1365-2958.1991.tb01849.x. 1809833

[34] Andreassen PR, Fredberg S, Horan M, Knudsen MH, Jacobsen K, Kjær A, Krogh TJ, Kronborg T, Mattsson NC, Schmidt SI, Wille H, Andersen A. 2014. Natural LacI from *E. coli* yields faster response and higher level of expression than the LVA-tagged LacI. ACS Synth Biol 3:949–952. doi:10.1021/sb500031h. 25524095

[35] Hector RE, Mertens JA, Nichols NN. 2019. Development and characterization of vectors for tunable expression of both xylose-regulated and constitutive gene expression in *Saccharomyces* yeasts. N Biotechnol 53:16–23. doi:10.1016/j.nbt.2019.06.006. 31228662

[36] Weng Y, Yao J, Sparks S, Wang KY. 2017. Nattokinase: an oral antithrombotic agent for the prevention of cardiovascular disease. Int J Mol Sci 18:523. doi:10.3390/ijms18030523. 28264497 PMC5372539

[37] Guan C, Cui W, Cheng J, Zhou L, Liu Z, Zhou Z. 2016. Development of an efficient autoinducible expression system by promoter engineering in *Bacillus subtilis*. Microb Cell Fact 15:66. doi:10.1186/s12934-016-0464-0. 27112779 PMC4845504

[38] Cai D, Zhu C, Chen S. 2017. Microbial production of nattokinase: current progress, challenge and prospect. World J Microbiol Biotechnol 33:84. doi:10.1007/s11274-017-2253-2. 28378222

[39] Liu Z, Zheng W, Ge C, Cui W, Zhou L, Zhou Z. 2019. High-level extracellular production of recombinant nattokinase in *Bacillus subtilis* WB800 by multiple tandem promoters. BMC Microbiol 19:89. doi:10.1186/s12866-019-1461-3. 31064343 PMC6505213

[40] Fu G, Yue J, Li D, Li Y, Lee SY, Zhang D. 2022. An operator-based expression toolkit for *Bacillus subtilis* enables fine-tuning of gene expression and biosynthetic pathway regulation. Proc Natl Acad Sci USA 119:e2119980119. doi:10.1073/pnas.2119980119. 35263224 PMC8931375

[41] Keasling JD. 2012. Synthetic biology and the development of tools for metabolic engineering. Metab Eng 14:189–195. doi:10.1016/j.ymben.2012.01.004. 22314049

[42] Blazeck J, Alper HS. 2013. Promoter engineering: recent advances in controlling transcription at the most fundamental level. Biotechnol J 8:46–58. doi:10.1002/biot.201200120. 22890821

[43] Guiziou S, Sauveplane V, Chang HJ, Clerté C, Declerck N, Jules M, Bonnet J. 2016. A part toolbox to tune genetic expression in *Bacillus subtilis*. Nucleic Acids Res 44:7495–7508. doi:10.1093/nar/gkw624. 27402159 PMC5009755

[44] Li T, Li T, Ji W, Wang Q, Zhang H, Chen GQ, Lou C, Ouyang Q. 2016. Engineering of core promoter regions enables the construction of constitutive and inducible promoters in *Halomonas* sp. Biotechnol J 11:219–227. doi:10.1002/biot.201400828. 26332342

[45] Liu D, Mao Z, Guo J, Wei L, Ma H, Tang Y, Chen T, Wang Z, Zhao X. 2018. Construction, model-based analysis, and characterization of a promoter library for fine-tuned gene expression in *Bacillus subtilis*. ACS Synth Biol 7:1785–1797. doi:10.1021/acssynbio.8b00115. 29944832

[46] Helmann JD. 1995. Compilation and analysis of *Bacillus subtilis* σ^A^-dependent promoter sequences: evidence for extended contact between RNA polymerase and upstream promoter DNA. Nucleic Acids Res 23:2351–2360. doi:10.1093/nar/23.13.2351. 7630711 PMC307037

[47] Yu X, Xu J, Liu X, Chu X, Wang P, Tian J, Wu N, Fan Y. 2015. Identification of a highly efficient stationary phase promoter in *Bacillus subtilis*. Sci Rep 5:18405. doi:10.1038/srep18405. 26673679 PMC4682092

[48] Chen Y, Ho JML, Shis DL, Gupta C, Long J, Wagner DS, Ott W, Josić K, Bennett MR. 2018. Tuning the dynamic range of bacterial promoters regulated by ligand-inducible transcription factors. Nat Commun 9:64. doi:10.1038/s41467-017-02473-5. 29302024 PMC5754348

[49] Phan TT, Nguyen HD, Schumann W. 2006. Novel plasmid-based expression vectors for intra- and extracellular production of recombinant proteins in *Bacillus subtilis*. Protein Expr Purif 46:189–195. doi:10.1016/j.pep.2005.07.005. 16125412

[50] Hector RE, Mertens JA. 2017. A synthetic hybrid promoter for xylose-regulated control of gene expression in *Saccharomyces* yeasts. Mol Biotechnol 59:24–33. doi:10.1007/s12033-016-9991-5. 28012062

[51] Chen W, Zhang Y, Yeo WS, Bae T, Ji Q. 2017. Rapid and efficient genome editing in *Staphylococcus aureus* by using an engineered CRISPR/Cas9 system. J Am Chem Soc 139:3790–3795. doi:10.1021/jacs.6b13317. 28218837

[52] Han L, Liu X, Cheng Z, Cui W, Guo J, Yin J, Zhou Z. 2022. Construction and application of a high-throughput in vivo screening platform for the evolution of nitrile metabolism-related enzymes based on a desensitized repressive biosensor. ACS Synth Biol 11:1577–1587. doi:10.1021/acssynbio.1c00642. 35266713

[53] Liu Z, Yu L, Zhou L, Zhou Z. 2019. One-pot biosynthesis of l-aspartate from maleate via an engineered strain containing a dual-enzyme system. Appl Environ Microbiol 85:e01327-19. doi:10.1128/AEM.01327-19. 31324629 PMC6752015

