# Supplementary material for: Development of a Glycerol-Inducible Expression System for High-Yield Heterologous Protein Production in Bacillus subtilis
Source: Microbiol Spectr. 2022 Aug 29;10(5):e01322-22. doi: 10.1128/spectrum.01322-22 (PMC9604022; doi:10.1128/spectrum.01322-22)
Supplement: Supplemental file 1 — Fig. S1-S6, Tables S1-S4. Download spectrum.01322-22-s0001.pdf, PDF file, 2.2 MB [file spectrum.01322-22-s0001.pdf]

Supplemental Material

Development of a glycerol-inducible expression system for high yield heterologous protein production in *Bacillus subtilis*

Laichuang Han, Qiaoqing Chen, Jie Luo, Wenjing Cui, Zhemin Zhou*

School of Biotechnology, Jiangnan University, Wuxi, Jiangsu, China

*Corresponding author

Zhemin Zhou,

School of Biotechnology, Jiangnan University

Wuxi, Jiangsu, 214122, China

E-mail: zhmzhou@jiangnan.edu.cn

Contents

Figures

Figure S1 Structure of GlpP-regulated promoters. 3

Figure S2 Comparison of the activities of GlpP-regulated promoters. 4

Figure S3 SDS-PAGE analysis of glycerol dose-dependent sfGFP expression by GIES. 5

Figure S4 Verification of the GlpP function. 6

Figure S5 The 96-well plate screening for the random mutation library of the -10 box of P_{glpD}. 7

Figure S6 Plasmid map. 8

Tables

Table S1 Plasmids and strains 9

Table S2 Primers 11

Table S3 Mutants of P_{glpD} 13

Table S4 Sequences of hybrid promoters 14

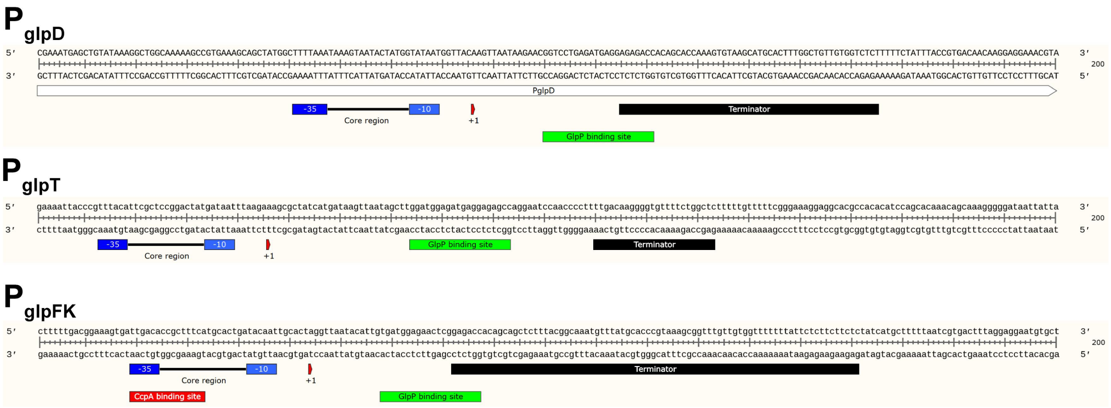

Figure S1 Structure of GlpP-regulated promoters.

The "-35" box and "-10" box were labeled by blue and light blue, respectively. The binding site of positive regulator, GlpP only here, was labeled by green. The binding site of negative regulator, CcpA only here, was labeled by red. The inherent terminator was labeled by black. All sequence annotations are according to the DBTBS database (https://dbtbs.hgc.jp/)[1].

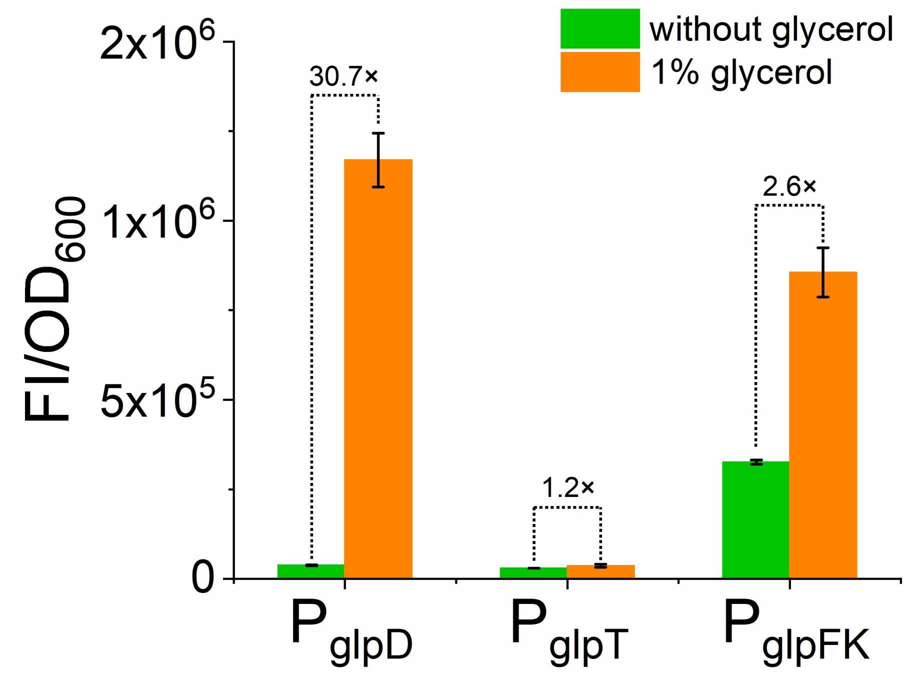

Figure S2 Comparison of the activities of GlpP-regulated promoters.

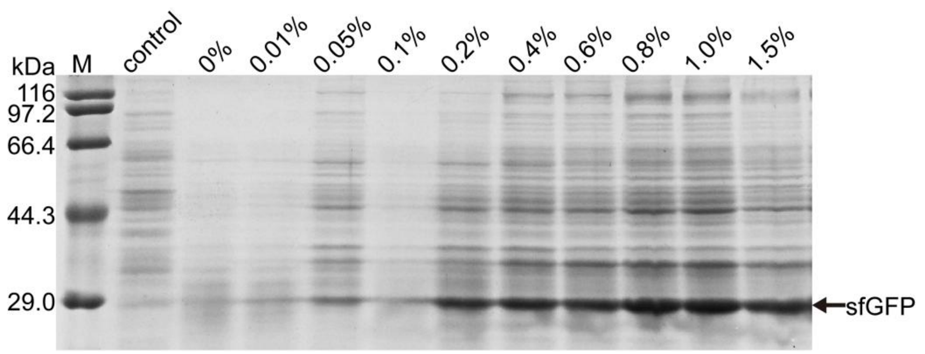

Figure S3 SDS-PAGE analysis of glycerol dose-dependent sfGFP expression by GIES.

Control: *B. subtilis* 168 without plasmid.

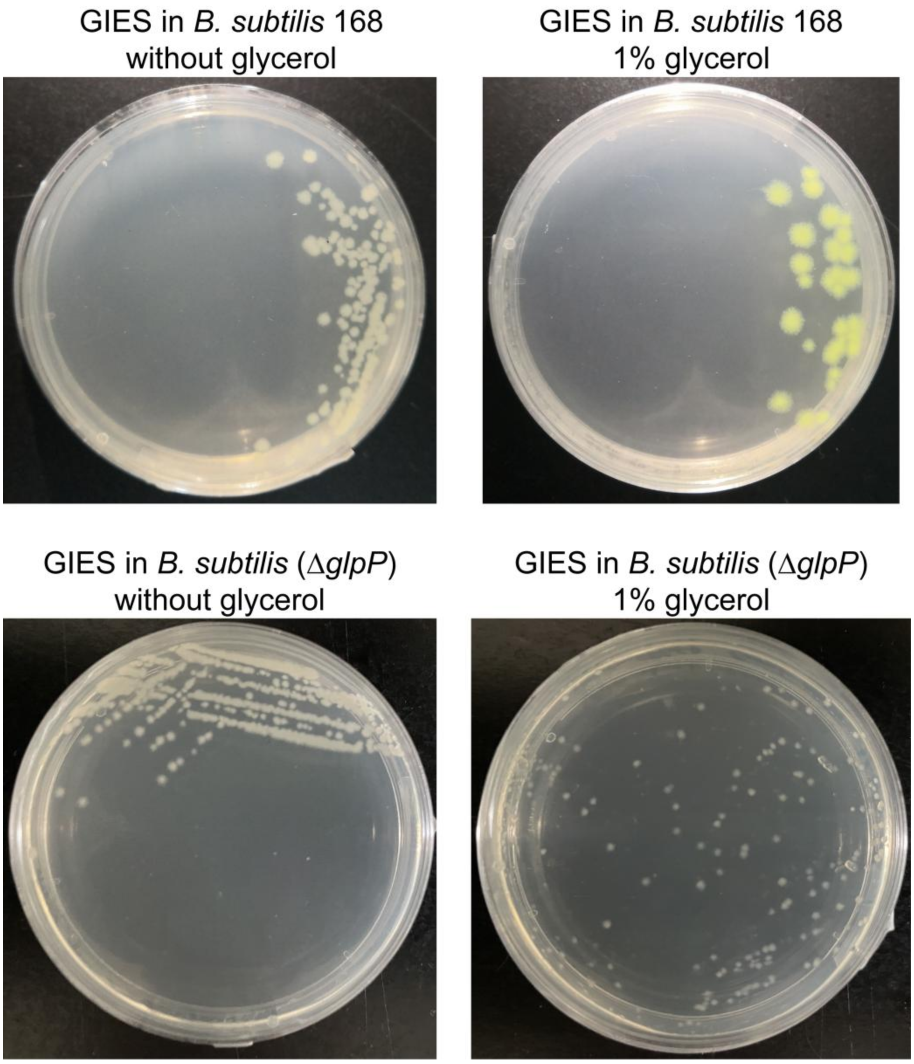

Figure S4 Verification of the GlpP function.

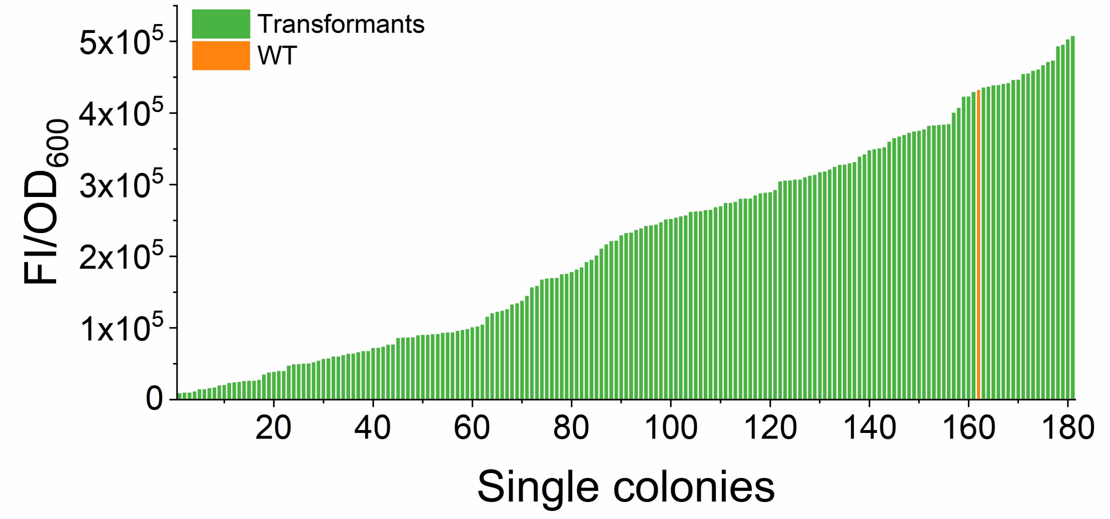

Figure S5 The 96-well plate screening for the random mutation library of the -10 box of P_{glpD}.

The concentration of glycerol used for the induction of sfGFP expression was 1% ($w \cdot v^{-1}$). Results are ranked according to FI/OD_{600} from lowest to highest.

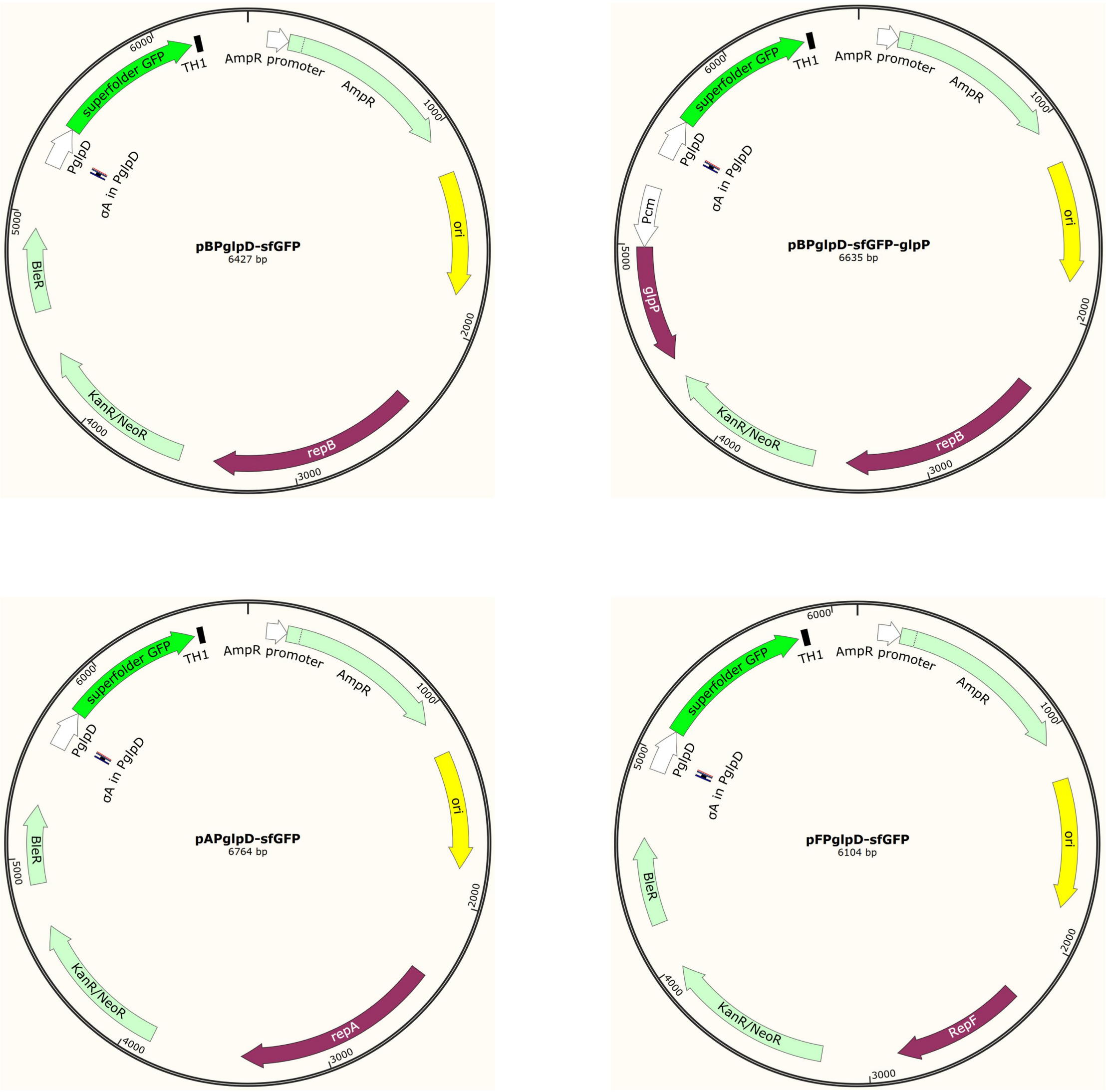

Figure S6 Plasmid map.

Table S1 Plasmids and strains

Plasmids and strains	Relevant characteristics	References or source
Plasmids		
pBP43-sfGFP	*E.coli*-*B.subtilis* shuttle vector, Amp^R in *Escherichia coli*, Kan^R in *Bacillus subtilis*, RepB, P43	Lab stork
pHT01	*E.coli*-*B.subtilis* shuttle vector, Amp^R in *Escherichia coli*, Cm^R in *Bacillus subtilis*, RepA, P_{grac}	Lab stork
pCasSA	Cas9，RepF，KanR，CmR	Addgene[2]
pBPglpD-sfGFP	Derived from pBP43-sfGFP, P43 was replaced with P_{glpD}	This study
pBPglpT-sfGFP	Derived from pBP43-sfGFP, P43 was replaced with P_{glpT}	This study
pBPglpFK-sfGFP	Derived from pBP43-sfGFP, P43 was replaced with P_{glpFK}	This study
pAPglpD-sfGFP	Derived from pBPglpD-sfGFP, RepA	This study
pFPglpD-sfGFP	Derived from pBPglpD-sfGFP, RepF	This study
pBPglpD-sfGFP-GlpP	Derived from pBPglpD-sfGFP, the GlpP expression cassette was introduced	This study
pBPglpD-AspA	Derived from pBPglpD-sfGFP, sfGFP was replaced by the *aspA* gene from *E.coli*	This study
pBPglpD-AspA-GlpP	Derived from pBPglpD-sfGFP-GlpP, sfGFP was replaced by the *aspA* gene from *E.coli*	This study
pBPglpD-NK-GlpP	Derived from pBPglpD-sfGFP-GlpP, sfGFP was replaced by the NK gene	This study
pBP43-glpD-sfGFP	Derived from pBPglpD-sfGFP, sfGFP was expressed under control of the hybrid promoter P43-glpD	This study
pBPsucA-glpD-sfGFP	Derived from pBPglpD-sfGFP, sfGFP was expressed under control of the hybrid promoter P_{sucA}-glpD	This study

pBPylbP-glpD-sfGFP	Derived from pBPglpD-sfGFP, sfGFP was expressed under control of the hybrid promoter P_{ylbP}-glpD	This study
pBPveg-glpD-sfGFP	Derived from pBPglpD-sfGFP, sfGFP was expressed under control of the hybrid promoter P_{veg}-glpD	This study
Strains		
E. coli JM109	*recA*1, *supE*44 *endA*1 *hsdR*17 ($^{r-}$k,m^{+} k) *gyrA*96 *relA*1 thi (*lac-proAB*) F'[*traD*36 *proAB*$^{+}$ *lacI*^{q} *lacZ* ΔM15]	Lab stork
B. subtilis 168	*trpC2*	Lab Stock
B. subtilis(DglpP)	Derive from *B. subtilis* comK*,glpP::Bleo,*Amp^{R}	
B. subtilis WB800	*168, DnprE, DaprA, Depr, Dbpr, Dmpr, DnprB, Dvpr, DwprA*	Lab Stock
BS-P43-sfGFP	*B.subtilis 168* harboring plasmid pBP43-sfGFP	This study
BPglpD-sfGFP	*B.subtilis 168* harboring plasmid pBPglpD-sfGFP	This study
APglpD-sfGFP	*B.subtilis 168* harboring plasmid pAPglpD-sfGFP	This study
FPglpD-sfGFP	*B.subtilis 168* harboring plasmid pFPglpD-sfGFP	This study
SGIES-sfGFP	*B.subtilis 168* harboring plasmid pBPglpD-sfGFP-GlpP	This study
GIES-AspA	*B.subtilis 168* harboring plasmid pBPglpD-AspA	This study
SGIES-AspA	*B.subtilis 168* harboring plasmid pBPglpD-AspA-GlpP	This study
WB-SGIES-NK	*B.subtilis 168* harboring plasmid pBPglpD-NK-GlpP	This study

Table S2 Primers

Primers	Sequences (5'-3')
PglpD-sfGFP-i1	GAGGGCAGGTCGAAATGAGCTGTATAAAGGC
PglpD-sfGFP-i2	CTCCTTTGCTCATTACGTTTCCTCCTTGTTGTCAC
PglpD-sfGFP-v1	GGAGGAAACGTAATGAGCAAAGGAGAAGAACTTTTC
PglpD-sfGFP-v2	ATACAGCTCATTTCGACCTGCCCTCTGCCACC
PglpT-sfGFP-i1	AGGTGGCAGAGGGCAGGTGAAAATTACCCGTTTACATTCGC
PglpT-sfGFP-i2	AGTTCTTCTCCTTTGCTCATTAATAATTATCCCCCTTTGCTGTTTG
PglpFK-sfGFP-i1	AGGTGGCAGAGGGCAGGTCTTTTTGACGGAAAGTGATTGAC
PglpFK-sfGFP-i2	AGTTCTTCTCCTTTGCTCATAGCACATTCCTCCTAAAGTCACG
PA-glpD-v1	ATACAAATTACATTTAACAGTTAAGTATTTATTTC
PA-glpD-v2	ACCTGCCCTCTGCCACCTC
PA-glpD-i1	TGAGGTGGCAGAGGGCAGGTCGAAATGAGCTGTATAAAGGC
PA-glpD-i2	CTGTTAAATGTAATTTGTATAAAAAAATACCCTCCTAGTACAG
PF-glpD-v1	TTTCTCCTTACGCATCTGTGCG
PF-glpD-v2	ACCTGCCCTCTGCCACCTC
PF-glpD-i1	TGAGGTGGCAGAGGGCAGGTCGAAATGAGCTGTATAAAGGC
PF-glpD-i2	CACAGATGCGTAAGGAGAAAAAAAAAATACCCTCCTAGTAC
PglpD-GlpP-i1	TAACGGAATATCAATCACTTTCCGTCAAAAAG
PglpD-GlpP-i2	TAGGAGGCATATCAAATGATGAGTTTTCACAACCAGCC
PglpD-GlpP-v1	TCGTCCATTCCGTTTCGGTAAGCTAGACAAAACG
PglpD-GlpP-v2	ACGGAAAGTGATTGATATTCCGTTAATGCGCCATG
PglpD-GlpP-p1	AACTCATCATTTGATATGCCTCCTAAATTTTTATCTAAAG
PglpD-GlpP-p2	TAGCTTACCGAAACGGAATGGACGATCGGCAATAG
P-AspA-i1	GACAACAAGGAGGAAACGTAATGTCAAACAACATTCGTATCG
P-AspA-i2	ATACCGCATCAGGCGAATTCTTACTGTTCGCTTTCATCAGTATAGC
P-AspA-v1	GAATTCGCCTGATGCGGTATC
P-AspA-v2	TACGTTTCCTCCTTGTTGTCACG
P-NK-i1	AGGAAACGTAATGAAAAAAAGAAAGAGGCGAAAC

P-NK-i2	GAGGGATACCGCATCAGGCGAATTCTTATTGTGCAGCTGCTTGTACG
P-NK-v1	GAATTCGCCTGATGCGG
P-NK-v2	CTTTCTTTTTTTCATTACGTTTCCTCCTTGTTGTCAC
PPglpD-10-1	GCTTTTAAATAAAGTAATACTATGGNNNNNNGGTTACAAGTTAATAAGAACGGTCC
PPglpD-10-2	CCATAGTATTACTTTATTTAAAAGCCATAGC
PP43-glpD-i1	TGGCAGAGGGCAGGTTGATAGGTGGTATGTTTTCGCTTG
PP43-glpD-i2	CTTATTAACTTTTATATTTTACATAATCGCGCGC
PP43-glpD-v1	TAAAATATAAAGTGATAGCGGAGTTAATAAGAACGGTCC
PP43-glpD-v2	TAACTCCGCTATCACTTTATATTTTACATAATCGCGCGC
PPsucA-glpD-1	AACAATCAAGGTAGAATCAAATTGCAAACAGTGGTAAAATATAGTTAATAAGAACGGTCCTGAG
PPsucA-glpD-2	GCAATTTGATTCTACCTTGATTGTTCACAAAATAGTAAAAAACACCTGCCCTCTGCCACCTC
PPylbP-glpD-1	TTAAATATTTGGATTTTTTAAATAAAGCGTTTACAATATATGTAGAAACAACAAAGTTAATAAGAACGGTCCTGAG
PPylbP-glpD-2	TTATTTAAAAAATCCAAATATTTAAACTTTAATTTTAAGCACATGGGATCTTTGAGAAGTACCTGCCCTCTGCCACCTC
PPveg-glpD-1	TTTATTTGACAAAAATGGGCTCGTGTTGTGCAATAAATGTAGTGAGGTGGAGTTAATAAGAACGGTCC
PPveg-glpD-2	CACGAGCCCATTTTTGTCAAATAAAATTTAAATTATATCAACGTTACCTGCCCTCTGCCACCTC

Note: N stands for 4 base (A, T, C, G) with same probability.

Table S3 Mutants of P_{glpD}

Mutants of P_{glpD}	Sequences of -10 box (5’-3’)
WT	TATAAT
AB4	ACAACG
BF9	TAGTGT
AF5	TAGATT
BC4	GATAAT
AA3	TAAAGT
BF10	TAAGCT
AE5	TAATAT
AD3	TAAGAT

Table S4 Sequences of hybrid promoters

Hybrid promoters	Sequences (5’-3’)
P43-glpD	TGATAGGTGGTATGTTTTCGCTTGAACTTTTAAATACAGCCATTGA<br>ACATACGGTTGATTTAATAACTGACAAACATCACCCTCTTGCTAAA<br>GCGGCCAAGGACGCTGCCGCCGGGGCTGTTTGCGTTTTTGCCGT<br>GATTTCGTGTATCATTGGTTTACTTATTTTTTTGCCAAAGCTGTAAT<br>GGCTGAAAATTCTTACATTTATTTTACATTTTTAGAAATGGGCGTG<br>AAAAAAAGCGCGATTATGTAAAATATAAAGTGATAGCGGAGTT<br>AATAAGAACGGTCCTGAGATGAGGAGAGACCACAGCACCAAAG<br>TGTAAGCATGCACTTTGGCTGTTGTGGTCTCTTTTTCTATTTACCG<br>TGACAACAAGGAGGAAACGTA
P_{sucA}-glpD	GTTTTTTACTATTTTGTGAACAATCAAGGTAGAATCAAATTGCAAA<br>CAGTGGTAAAATATAGTTAATAAGAACGGTCCTGAGATGAGGAGA<br>GACCACAGCACCAAAGTGTAAGCATGCACTTTGGCTGTTGTGGT<br>CTCTTTTTCTATTTACCGTGACAACAAGGAGGAAACGTA
P_{ylbP}-glpD	ACTTCTCAAAGATCCCATGTGCTTAAAATTAAAGTTTAAATATTTG<br>GATTTTTTAAATAAAGCGTTTACAATATATGTAGAAACAACAAAGT<br>TAATAAGAACGGTCCTGAGATGAGGAGAGACCACAGCACCAAAG<br>TGTAAGCATGCACTTTGGCTGTTGTGGTCTCTTTTTCTATTTACCG<br>TGACAACAAGGAGGAAACGTA
P_{veg}-glpD	AACGTTGATATAATTTAAATTTTATTTGACAAAAATGGGCTCGTGT<br>TGTGCAATAAATGTAGTGAGGTGGAGTTAATAAGAACGGTCCTGA<br>GATGAGGAGAGACCACAGCACCAAAGTGTAAGCATGCACTTTGG<br>CTGTTGTGGTCTCTTTTTCTATTTACCGTGACAACAAGGAGGAAA<br>CGTA

Note: The sequences of the 5’-UTR of P_{glpD} were colored by orange; the sequences of constitutive promoters were colored by blue; the transcription start site was labeled by underline.

References

1 Sierro, N., Makita, Y., de Hoon, M. & Nakai, K. DBTBS: a database of transcriptional regulation in Bacillus subtilis containing upstream intergenic conservation information. *Nucleic acids research* **36**, D93-96, doi:10.1093/nar/gkm910 (2008).

2 Chen, W., Zhang, Y., Yeo, W. S., Bae, T. & Ji, Q. Rapid and Efficient Genome Editing in Staphylococcus aureus by Using an Engineered CRISPR/Cas9 System. *Journal of the American Chemical Society* **139**, 3790-3795, doi:10.1021/jacs.6b13317 (2017).
